# Inhibition of BTK and ITK with Ibrutinib Is Effective in the Prevention of Chronic Graft-versus-Host Disease in Mice

**DOI:** 10.1371/journal.pone.0137641

**Published:** 2015-09-08

**Authors:** Steven D. Schutt, Jianing Fu, Hung Nguyen, David Bastian, Jessica Heinrichs, Yongxia Wu, Chen Liu, Daniel G. McDonald, Joseph Pidala, Xue-Zhong Yu

**Affiliations:** 1 Department of Microbiology & Immunology, Medical University of South Carolina, Charleston, South Carolina, United States of America; 2 Cancer Biology PhD Program, University of South Florida and H. Lee Moffitt Cancer Center, Tampa, Florida, United States of America; 3 Pathology, Immunology and Laboratory Medicine, University of Florida, Gainesville, Florida, United States of America; 4 Department of Radiation Oncology, Medical University of South Carolina, Charleston, South Carolina, United States of America; 5 Blood and Bone Marrow Transplant Department, Moffitt Cancer Center, Tampa, Florida, United States of America; 6 Department of Medicine, Medical University of South Carolina, Charleston, South Carolina, United States of America; University of Toledo, UNITED STATES

## Abstract

Bruton’s Tyrosine Kinase (BTK) and IL-2 Inducible T-cell Kinase (ITK) are enzymes responsible for the phosphorylation and activation of downstream effectors in the B-cell receptor (BCR) signaling and T cell receptor (TCR) signaling pathways, respectively. Ibrutinib is an FDA-approved potent inhibitor of both BTK and ITK that impairs B-cell and T-cell function. CD4 T cells and B cells are essential for the induction of chronic graft-versus-host disease (cGVHD). We evaluated these targets by testing the ability of Ibrutinib to prevent or ameliorate cGVHD, which is one of the major complications for patients undergoing allogeneic hematopoietic stem cell transplantation (allo-HSCT). We found that Ibrutinib significantly alleviated cGVHD across four different mouse models, accompanied by increased long-term survival and reduced clinical score. The clinical improvements in Ibrutinib-treated recipients were associated with decreased serum-autoantibodies, costimulatory molecule activation, B-cell proliferation, and glomerulonephritis compared to vehicle controls. Ibrutinib was also able to alleviate the clinical manifestations in acute GVHD (aGVHD), where the recipients were given grafts with or without B cells, suggesting that an inhibitory effect of Ibrutinib on T cells contributes to a reduction in both aGVHD and cGVHD pathogenesis. An effective prophylactic regimen is still lacking to both reduce the incidence and severity of human cGVHD following allo-HSCT. Our study shows that Ibrutinib is an effective prophylaxis against several mouse models of cGVHD with minimal toxicity and could be a promising strategy to combat human cGVHD clinically.

## Introduction

Chronic graft-versus-host disease (cGVHD) is a lethal complication following allogeneic hematopoietic stem cell transplantation (allo-HSCT) with autoimmune-like manifestations [1]. Effective prophylactic and post-allo-HSCT therapies for cGVHD are still lacking [2, 3]. CD4^+^ T cells and B cells are the essential components that contribute to cGVHD pathogenesis, causing a cascade of T- and B-cell priming, activation, expansion, autoantibody production, migration, and tissue damage via inflammation and fibrosis [1, 4]. T- and B-cell dysregulation leading to excessive and uncontrolled pathogenic antibody production is a primary feature of cGVHD found in both humans and mouse models [4–7]. Activation of both B-cell receptor (BCR) and T- cell receptor (TCR) signaling pathways and the resulting costimulation of B cells by T cells are essential for cGVHD induction and development, which has warranted investigation into potential therapeutic targets in these pathways [8, 9].

Bruton’s Tyrosine Kinase (BTK) and IL-2 Inducible T-cell Kinase (ITK) are BCR and TCR signaling complexes, respectively [10–12]. Although the distinct involvement of BTK and ITK in the induction and pathogenesis of cGVHD is not known, BTK is largely responsible for B-cell differentiation, activation, and the initiation of autoantibody production [13, 14] whereas ITK is primarily involved in the secretion of IL-2 and Th2 cytokines [15] while also affecting the balance between Th17 and T regulatory cells (Tregs) [16]. Ibrutinib (PCI-32765) is an FDA-approved potent irreversible inhibitor of both BTK and ITK [17] for treatment of chronic lymphocytic leukemia (CLL) involving unchecked B cell growth and expansion [18]. In addition, Ibrutinib has recently been FDA-approved for treatment of Waldenström’s macroglobulinemia, a lymphoproliferative cancer.

Current mouse models of cGVHD are limited in that each model can only produce a portion of the human clinical manifestations such as autoantibody production, scleroderma, loss of thymic function, and multiple organ system inflammation including fibrosis [19]. We, therefore, utilized several distinct mouse models of cGVHD to test the prophylactic potential of Ibrutinib. The DBA/2→BALB/c model involves a transition between aGVHD and a sclerotic form of cGVHD including Ig autoantibody deposition into target tissues measurable by albumin protein levels and ELISA [4]. The DBA/2→B6D2F1 model produces a stronger autoantibody response in reaction to the allogeneic transplant causing significant Ig deposits into target tissues such as the kidneys [20]. The B10.D2→BALB/c model produces extensive sclerodermatous skin damage in the recipients, which is a primary clinical feature of human cGVHD [21]. In the B6→BALB/c model of cGVHD, severe gut, skin, and thymic pathological damage is produced in recipients in addition to autoantibody production [5]. Additionally, two models of aGVHD (B6.129S2-*Ighmtm1Cgn*/J→BALB/c and B6→BALB/c) were used to test the effect of Ibrutinib on donor T cells via ITK inhibition in the absence or presence of donor B cells in the graft, respectively.

## Material and Methods

This study was carried out in strict accordance with the recommendations in the Guide for the Care and Use of Laboratory Animals of the National Institutes of Health. This specific study was included in a protocol that was sent to and approved by the Institutional Animal Care and Use Committee of the Medical University of South Carolina (Permit Number: 3221). Isoflurane was used in all procedures involving pain or distress. All efforts were made to minimize suffering.

### Mice

DBA/2 (H-2^d^), B6D2F1 (H-2^b/d^), BALB/c (H-2^d^), C57BL/6 (B6; H-2^b^, CD45.2), and B6.Ly5.1 (CD45.1) mice were purchased from National Cancer Institute (NCI, Frederick, MD).

B10.D2 (H-2^d^) and B6.129S2-*Ighmtm1Cgn*/J (B-cell deficient; BKO B6) mice were purchased from the Jackson Laboratory (Bar Harbor, ME). All animals were housed in the American Association for Laboratory Animal Care–accredited Animal Resource Center at Medical University of South Carolina (MUSC, Charleston, SC).

### cGVHD models

Distinct major histocompatibility complex (MHC)-matched but minor histocompatibility antigen (miHA)–mismatched mouse models of cGVHD (DBA2→BALB/c, DBA2→B6D2F1, and B10.D2→BALB/c), or both MHC and miHA-mismatched model (B6Ly5.1^+^→BALB/c) were used to test the efficacy of Ibrutinib. T cell–depleted bone marrow cells, referred to as TCD-BM, were obtained as previously described [22], and used in all conditioning-dependent transplantation experiments throughout. CD25-depleted splenocytes were obtained by negative selection after incubation with biotin-conjugated anti-CD25 antibody (eBioscience), and subsequently anti-biotin magnetic beads, according to the manufacturer's instructions (Miltenyi Biotech, San Diego, CA).

BALB/c recipients (8–10 week old) were conditioned with total body irradiation (TBI) at 700 cGy (single dose) using an RAD 320 X-ray Irradiator (Precision X-ray Inc., North Branford, CT). Irradiated recipients were intravenously injected with 5 x 10^6^ TCD-BM alone or with 40 x 10^6^ CD25^-^ splenocytes (DBA/2→BALBc); 5 x 10^6^ whole splenocytes (B10.D2→BALB/c); 1–2 x 10^6^ whole splenocytes (B6Ly5.1^+^→BALB/c) from respective donors within 24 hours post-conditioning. B6D2F1 recipients (8–10 week old) were unconditioned, and were intravenously injected with 80–100 x 10^6^ whole splenocytes from the donors or received no injection as a control (DBA/2→B6D2F1).

Recipient mice were monitored for weight loss, proteinuria using Albustix test strips (Siemens Corporation, Washington, D.C., USA), and other clinical signs of cGVHD once a week. Mice were considered as having developed proteinuria when the albumin protein level measured by the Alubstix test strips was ≥ 2000 mg/dl as described previously [4]. Clinical scores were tabulated as 7 parameters optimized from a previous report [23]: weight loss, posture, activity, fur texture, skin integrity, diarrhea, and eye inflammation or conjunctivitis. Individual mice were scored 0 to 2 for each criteria and 0 to 12 overall. We arbitrarily considered mice falling under the score categories 0.5–3 as mild, 4–7 as moderate, and 8–12 as showing severe symptoms where scores ≥ 8 required euthanasia as a humane endpoint. Recipients reaching pre-moribund stage described in our IACUC-approved protocol were euthanized and accounted for mortality. GVHD target organs were excised from recipients 4, 28, or 60 days post-BMT and subjected to histology staining with hematoxylin and eosin (H&E), pathology scoring, and FACS analysis as described in a previous study [24]. Skin pathology scores were tabulated as 5 parameters from a previous report [25]: dermal fibrosis, fat loss, epidermal thickening, follicular loss, and inflammation. Individual mice were scored 0 to 2 for each criteria and 0 to 10 overall.

### aGVHD model

T-cell purification from whole spleen of either wild-type (WT) or B-cell deficient (BKO) B6 donors was done by negative depletion magnetically [22]. BALB/c recipients (8–10 week old) were conditioned with TBI at 700 cGy (single dose), and intravenously injected with 5 x 10^6^ TCD-BM alone or plus 0.5–0.75 x 10^6^ purified T cells from either WT B6 or BKO B6 mice within 24 hours post-conditioning.

### Preparation of drug

Ibrutinib was provided by pharmacylics (Sunnyvale, CA). Trappsol (hydroxypropyl-β-cyclodextrin) was purchased from CTD Holdings (Alachua, FL) as the vehicle for Ibrutinib and was dissolved at a 1:10 ratio in Nanopure water. This solution was then acidified until pH < 3. After vehicle acidification, Ibrutinib powder was added and thoroughly mixed to the concentration of 1–1.6 mg/ml. The pH was then adjusted to between 6.0 and 8.0. Final solution was then filtered through a .22μm pore size SFCA syringe filter. An appropriate amount of vehicle solution and vehicle plus Ibrutinib was mixed to obtain calculated dosages at 5, 10, 15, or 20 mg/kg based on animal weight.

### *In vivo* carboxyfluorescein diacetate succinimidyl ester (CFSE) proliferation assay

CD25^-^ splenocytes obtained from DBA/2 mice were washed and resuspended at a concentration of 20 x 10^6^ cells/ml in phosphate-buffered saline (PBS). CFSE (Invitrogen, Molecular Probes, Inc., Eugene, OR, USA) in dimethylsulfoxide (DMSO) (5 mM) was added to the CD25^-^ splenocytes suspension to a final concentration of 2 μM. The cells were gently mixed and incubated at 37°C for 7 minutes. The staining was quenched by the addition of RPMI1640 media containing 10% fetal bovine serum (FBS), and the cells were washed two times and then used for intravenous injection. After 4 days, recipient spleens were excised and the cells were stained with different antibodies and analyzed by flow cytometry.

### Flow cytometry

Recipient splenocytes and thymocytes were isolated and stained for surface receptors and intracellular cytokines using standard flow cytometric protocols as previously described [22, 24, 26]. The following Abs were used for cell-surface staining: anti-CD4–FITC, or–V450, anti-CD8α–FITC, or–allophycocyanin-cy7, anti-B220–v450 (RA3-6B2), anti-CD80–FITC, anti- CD86–FITC, anti-CD40 APC, Biotin-anti-CD29 (β1 integrin), anti-CD229.1–Biotin or PE, anti- CD5.1-FITC, purchased from BD Biosciences. Intracellular staining was carried out using anti–IFN-γ–PE or Per-cp 5.5 (XMG1.2; BD Biosciences), anti–IL-17–allophycocyanin (17B7; eBioscience), anti–IL-4–PE (11B11; BD Pharmingen), anti–IL-5–PE (TRFK5; BD Pharmingen), anti-TNFα–PE, or PE-Cy7 (MP6-XT22; BD Pharmingen), anti-Foxp3–PE (FJK-16s; eBioscience), and the appropriate isotype controls. Cell isolates were analyzed using Diva software, LSR II (BD Biosciences,San Jose, CA), FACS Verse (BD Biosciences, San Jose, CA), and FlowJo (TreeStar, Ashland, OR).

### Serum autoantibody detection

Using DNA from calf thymus (Sigma, D1501), we made double-stranded DNA (dsDNA) using a protocol previously described [27]. High-binding ELISA plates (Costar, 3369) were then coated with a mixture containing 5μg/ml dsDNA overnight at 37°C. The plates were then blocked with PBS containing 1% BSA for 30 minutes. Following blocking, plates were washed several times with 0.05% tween-20 PBS. Serum was added at a 1:10 ratio in PBS containing 0.05% Tween and 1% BSA (PBS-T/BSA) and then diluted using 2-fold serial dilutions until 1:80. Plates were incubated at room temperature (RT) for 45 minutes and then washed. The HRP-conjugated secondary antibody (HRP-IgG or HRP- IgG2a, Southern Biotech) was then added at a 1:4000 ratio in PBS-T/BSA and incubated for 45 minutes at RT. Plates were then washed and 50μl of TMB Substrate (eBioscience) was added to each well. The reaction was stopped after 10 minutes using 50μl of 1M phosphoric acid and the plate was read at 450nm. Serum consisting of high titers of IgG and IgG2a autoantibodies was used as a positive control on each plate in addition to wells with no serum as negative controls. Plates were read by a Multiscan FC (Thermo Scientific, Waltham, MA) ELISA plate reader. HRP-Anti-IgG and HRP-anti-IgG2a were purchased from Southern Biotech (Birmingham, AL).

### Statistics

For comparison of recipient proteinuria development and survival among groups in GVHD experiments, a log-rank test was used to determine any statistical significance. To compare differences in GVHD clinical scores, fluorescence intensity of cell isolates, optical density of serum autoantibodies, albumin protein levels present in urine, and percentages of cell populations, a two-tailed Student *t* test was used to determine any statistical significance (p<0.05) unless otherwise stated.

## Results

### Ibrutinib improves survival and reduces proteinuria development

**Fig 1:**
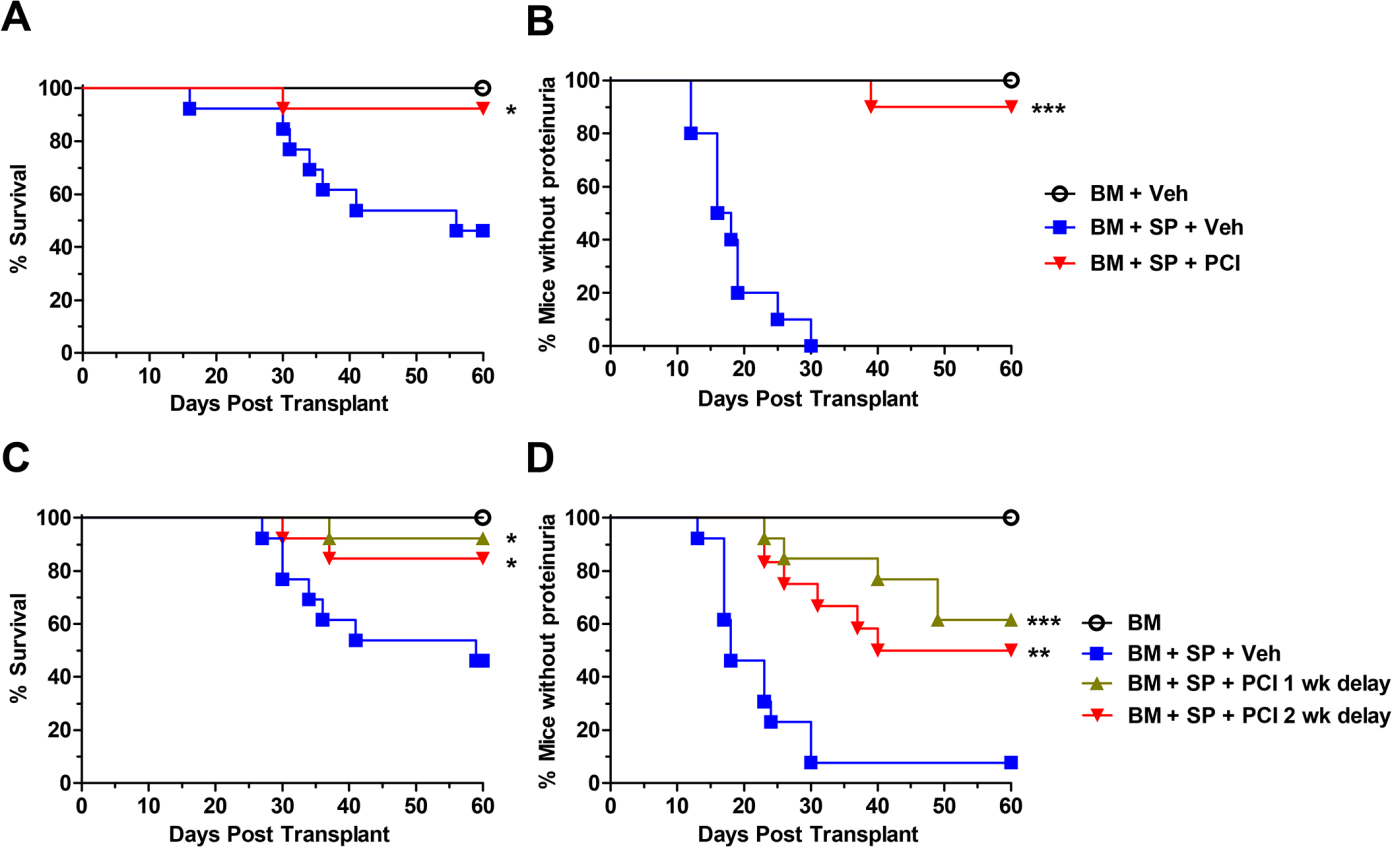
Ibrutinib improves survival and reduces glomerulonephritis induced proteinuria. Lethally irradiated BALB/c recipients were transplanted with TCD-BM (5 x 10^6^ per mouse) from DBA/2 donors with (n = 26) or without (n = 16) CD25^-^ splenocytes at 40 x 10^6^ per mouse. Groups were either given no treatment, daily oral gavage of vehicle alone, or Ibrutinib 10 mg/kg beginning 2–4 h before BMT and continued for 4 weeks. Mice were monitored for 60 d for survival (A) and proteinuria twice/week (B). Data are shown from two replicate experiments combined. In separate experiments under the same conditions as in A and B, daily oral gavage of vehicle (n = 13) and Ibrutinib treatment (n = 26) was delayed by either 1 (n = 13) or 2 (n = 13) weeks post-BMT and continued for 4 weeks. Mice were monitored for 60 d for survival (C) proteinuria twice/week (D). Data are shown from three replicate experiments combined. Asterisk indicates statistical significance between vehicle treatment and Ibrutinib treatment groups: *p<0.05, **p<0.01, ***p<0.001.

We first evaluated the effect of Ibrutinib in the development of cGVHD using a murine model involving both autoantibodies and scleroderma [4]. In DBA/2**→**BALB/c model after allo-BMT, recipients treated with vehicle control developed moderate (4–7 clinical scores) to severe (8–12 clinical scores) cGVHD symptoms as defined by the cGVHD clinical scoring system throughout the 60-day monitoring period. In severe cases, the disease led to kidney failure as determined by proteinuria and the development of ascites which included severe abdominal swelling, abdominal scleroderma, and eventually fatality. Recipients given Ibrutinib (abbreviated as PCI) as a prophylactic treatment displayed a significantly improved survival rate compared to vehicle controls (Fig 1A). Notably, Ibrutinib was also able to completely prevent ascites development in all subjects compared to vehicle controls (A in S1 Fig). Ibrutinib recipients showed significantly delayed onset and overall reduction of proteinuria development (Fig 1B). Additionally, we determined that the length of treatment was critical to fully suppress cGVHD induction, as 2-week treatment, in contrast to 4-week treatment, was not effective to prevent proteinuria development (B in S1 Fig).

To test the ability of Ibrutinib to reverse ongoing or already established cGVHD, we delayed treatment by either 1 or 2 weeks after allo-BMT. Despite the delay of treatment, the recipients treated with Ibrutinib still maintained a significantly higher rate of survival and significantly lower proteinuria development compared to those with vehicle controls (Fig 1C and 1D). Notably, prophylactic treatment of Ibrutinib was more effective in suppressing proteinuria development than the delayed treatment (Fig 1B and 1D).

### Ibrutinib reduces the proliferation of B cells, up-regulation of costimulatory molecules, and the production of serum autoantibodies

**Fig 2:**
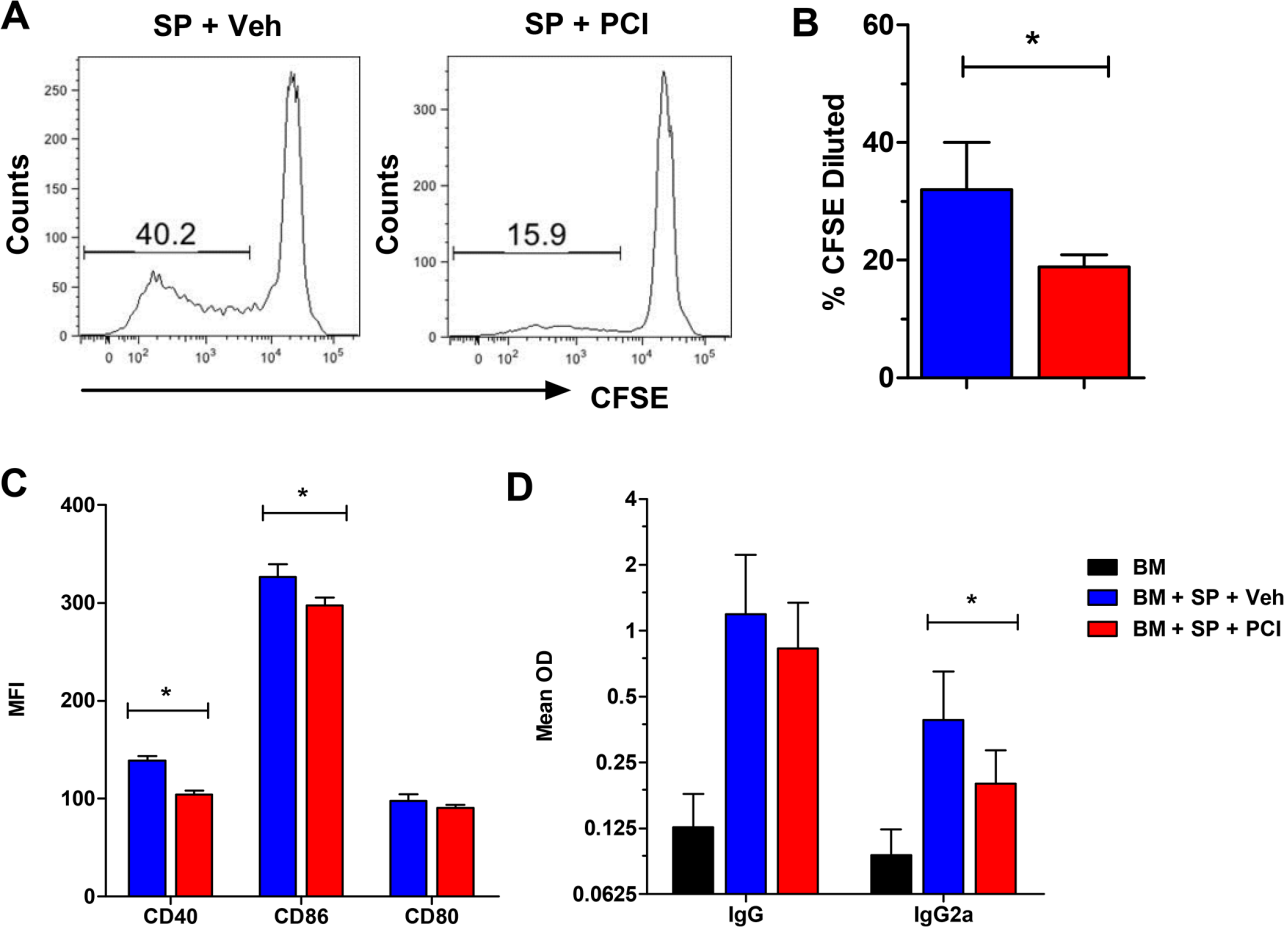
Ibrutinib reduces B-cell proliferation, costimulatory molecules, and dsDNA autoantibodies in cGVHD. Lethally irradiated BALB/c recipients were transplanted with or without CFSE labeled CD25^-^ splenocytes from DBA/2 donors at a dose of 40 x 10^6^ per mouse. Groups were either given no treatment (n = 6), daily oral gavage of vehicle alone (n = 12), or Ibrutinib 10 mg/kg (n = 12) beginning 2–4 hours before BMT and continued for 4 days. Recipient mice were euthanized 4 days after transplant and spleen was taken for FACS analysis. The percentage of CFSE dilution (A, B) represents the amount of proliferated donor B cells. The expression of CD40, CD80, and CD86 costimulatory molecules on B cells were analyzed by gating on B220^+^ cells and shown as mean florescence intensity (MFI) (C). D panel represents a separate experimental design where BMTs and the number of subjects were as described previously except recipients were sacrificed 28 days after BMT and the Ibrutinib treatment duration was 4 weeks. Serum from whole blood was taken on day 28 post-BMT for ELISA measuring autoantibodies IgG and IgG2a (D). Data are pooled from three replicate experiments. Asterisk indicates statistical significance: *p<0.05.

Using the DBA/2**→**BALB/c model, we performed a 4-day *in vivo* CFSE proliferation assay to determine the potential impact of Ibrutinib on early development of cGVHD. Compared to vehicle controls, recipients treated with Ibrutinib displayed significantly lower B-cell proliferation as measured by the percentage of diluted CFSE-labeled B220^+^ donor cells (Fig 2A and 2B). The recipients treated with Ibrutinib also displayed significantly lower expression of CD86 and CD40 costimulatory molecules on B cells (Fig 2C), which have been shown to correlate with cGVHD [28]. In separate experiments, we collected serum from peripheral blood on day 28 post-BMT. The treatment with Ibrutinib also significantly lowered the levels of IgG2a, but not total IgG, autoantibodies compared to vehicle controls (Fig 2D). In contrast to B cells, we did not observe a significant effect of Ibrutinib on T-cell proliferation using this cGVHD model (S2 Fig). After a monitoring period of 28 days post transplant, we also found that the absolute number of CD4 and CD8 double positive thymocytes was significantly increased in Ibrutinib treated recipients compared to vehicle controls (B in S3 Fig), suggesting reduced cGVHD pathogenesis and improved immune reconstitution [5]. Interestingly, we compared oral gavage and i.p. injection, and found that oral administration of Ibrutinib was significantly more effective at reducing cGVHD than i.p. injection as measured by albumin protein levels in the urine (A in S3 Fig).

### Ibrutinib reduces proteinuria and serum autoantibodies in DBA/2→B6D2F1 model

**Fig 3:**
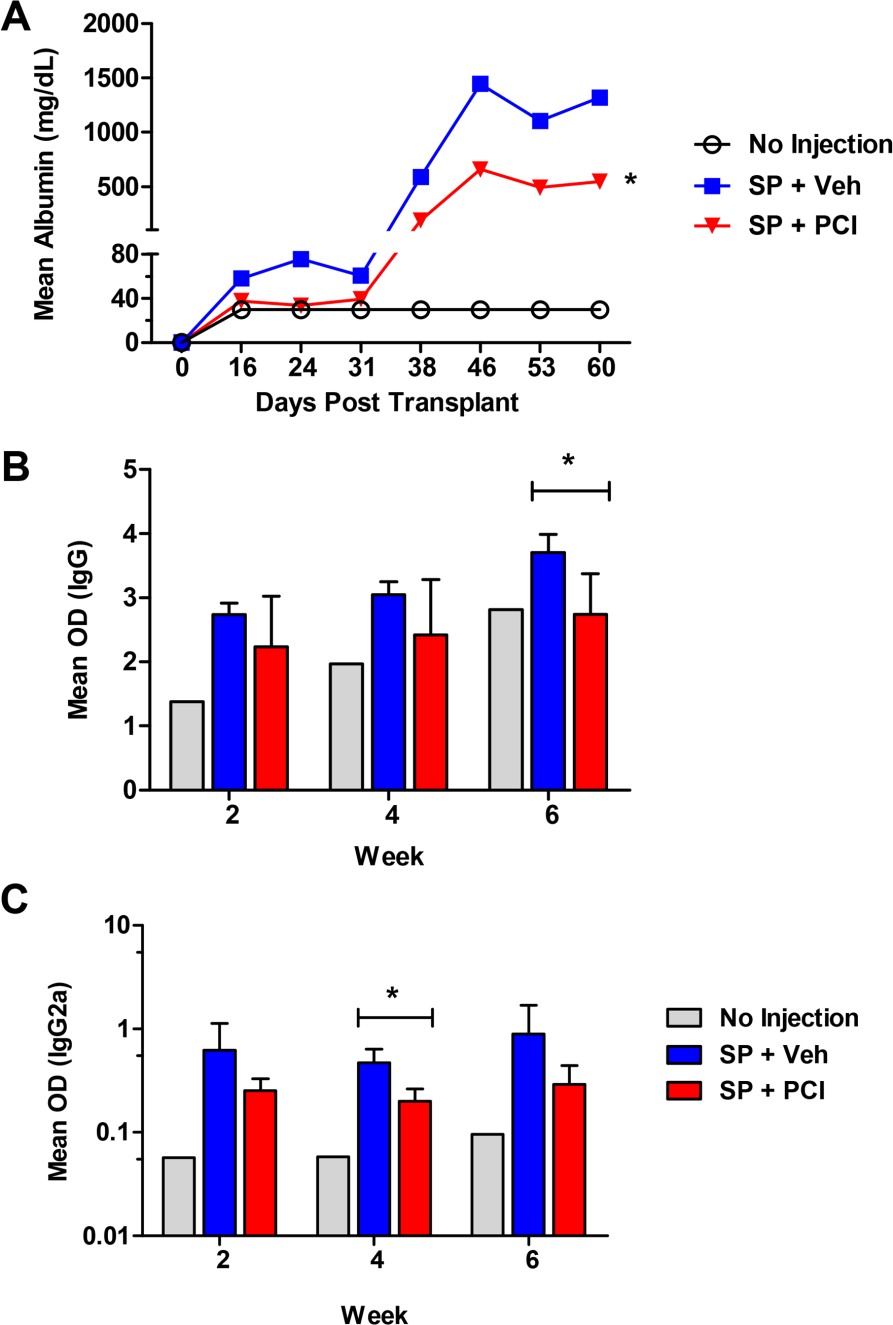
Ibrutinib reduces glomerulonephritis and dsDNA autoantibodies. Unconditioned B6D2F1 recipients were transplanted with 80–95 x 10^6^ splenocytes without BM from DBA/2 donors. Groups were either given no treatment (n = 6), daily oral gavage of vehicle (n = 15), or two different dosages of Ibrutinib, 10 mg/kg (n = 5) or 20mg/kg (n = 10) 2–4 hours before transplant and continued for 4 weeks. Animals were monitored for proteinuria (A). Serum from whole blood was taken bi-weekly to monitor levels of IgG (B) and IgG2a (C) autoantibodies. The data are pooled from three replicate experiments. Asterisk indicates statistical significance between vehicle treatment and Ibrutinib treatment groups: *p<0.05.

Complementary to the DBA/2**→**BALB/c model, we also tested the ability of Ibrutinib to reduce cGVHD in an autoantibody-mediated model, DBA/2**→**B6D2F1. Although this model did not present clinically visible symptoms, we were able to measure proteinuria development as well as find strong autoantibody responses as a result of allo-BMT. B6D2F1 recipients given Ibrutinib as a prophylactic treatment developed significantly less proteinuria compared to vehicle controls (Fig 3A). Ibrutinib treatment was also able to reduce both total IgG and IgG2a serum-autoantibodies in all time points measured, and with significance on weeks 6 and 4, respectively (Fig 3B and 3C).

### Ibrutinib affects clinical features of cGVHD in the B10.D2→BALB/c model

**Fig 4:**
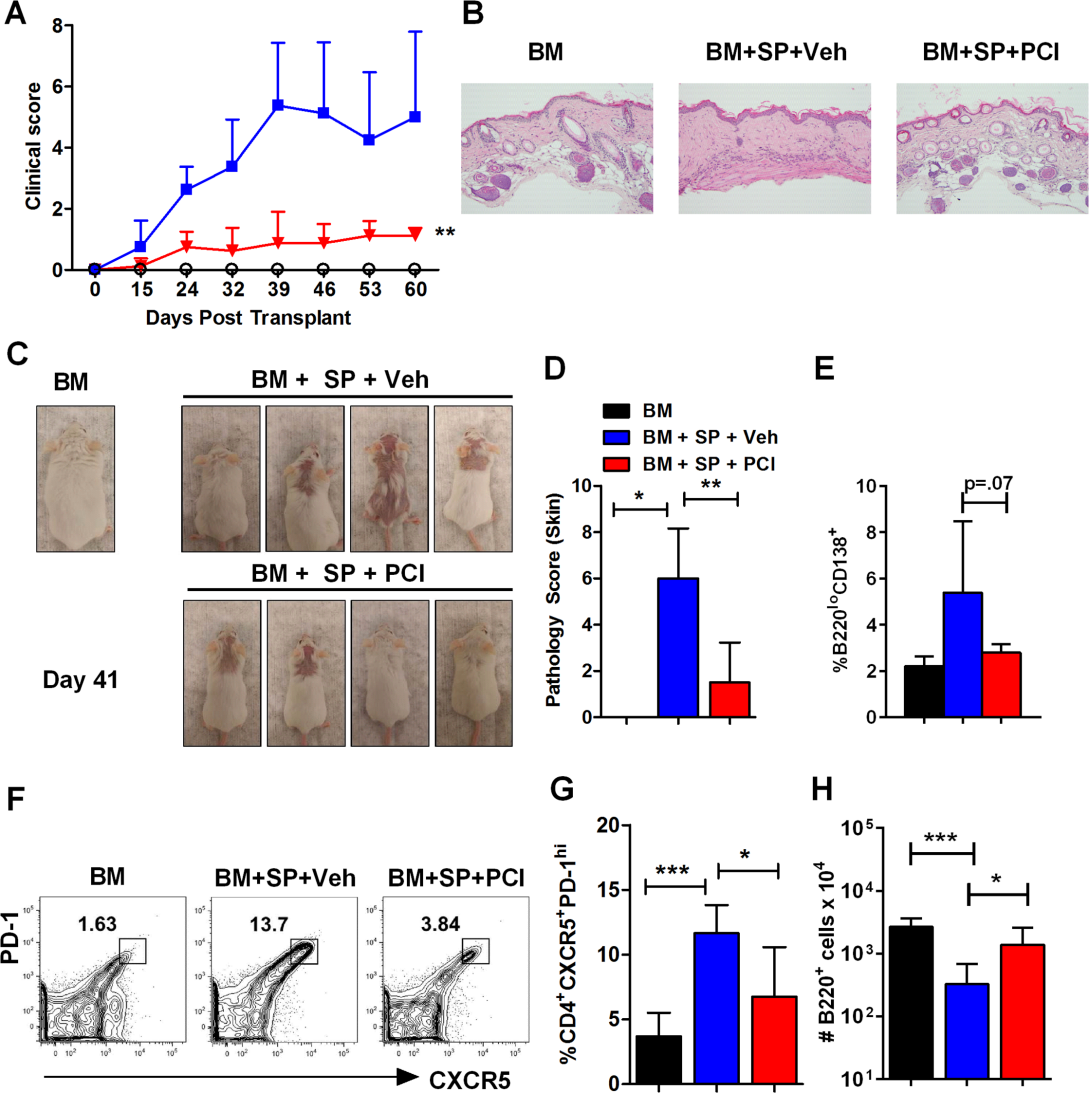
Ibrutinib delays onset and reduces severity of scleroderma development. Lethally irradiated BALB/c recipients were transplanted with TCD-BM (5 x 10^6^ per mouse) from B10.D2 donors with or without whole splenocytes at a dose of 5 x 10^6^ per mouse. Groups were either given no treatment (n = 4), daily oral gavage of vehicle alone (n = 9), or Ibrutinib at the dose of 10 mg/kg (n = 9) beginning 2–4 hours before BMT and continued for 4 weeks. Animals were monitored for survival and clinical score using a scoring system (A) and visual representations (C). At day 60, recipients were sacrificed and skin biopsies were taken for H&E staining (B) and scoring (D). Spleens were excised for FACS analysis (E-H). Percentage of B220^lo^CD138^+^ plasma cells were reported for BM, vehicle, and Ibrutinib treated groups (E). Representative flow plot (F) as well as the combined data (G) for the percentage of Tfh cells were shown. Absolute numbers of B220^+^ cells were calculated and presented (H). Data shown is pooled from two replicate experiments. Asterisk indicates statistical significance between vehicle treatment and Ibrutinib treatment groups: *p<0.05, **p<0.01, ***p<0.001.

Using a classical model of sclerodermatous cGVHD (B10.D2**→**BALB/c) [21], we tested the ability of Ibrutinib to ameliorate the primary feature of this model, skin damage, in addition to other clinical manifestations such as weight loss, diarrhea, eye dryness and eye inflammation. Using a cGVHD clinical score system, we found that administration of Ibrutinib (10mg/kg) prior to allo-BMT was able to significantly reduce cGVHD symptoms compared to vehicle controls (Fig 4A) in two replicate experiments where 7 of 9 recipients treated with Ibrutinib showed little to no signs of skin damage or other cGVHD symptoms. GVHD skin lesions were taken for H&E staining and the severity of the lesions were assessed by a professional pathologist; representative histology images are shown (Fig 4B). Notably, 2 of 9 recipients treated with Ibrutinib did develop moderate alopecia and redness but it was not characterized by scaling, dried blood, and other signs of sclerodermatous skin damage seen in the vehicle control groups (Fig 4C). Day 60 post-BMT skin biopsies taken from mice treated with 10mg/kg Ibrutinib showed significantly lower pathology scores compared to both the vehicle group (Fig 4C) and the 5mg/kg Ibrutinib treated group (S4 Fig). We also found that the treatment with 10 mg/kg Ibrutinib significantly improved B-cell reconstitution (Fig 4H), as well as reduced the percentage of CD4^+^CXCR5^+^PD-1^high^ T follicular helper cells (Tfh) (Fig 4F and 4G), compared to the vehicle treatment while not being significantly different than bone marrow alone controls. Additional experiments revealed that splenic autoantibody producing B220^lo^CD138^+^ plasma cells were significantly reduced by 10mg/kg Ibrutinib treatment compared to a lower dose of Ibrutinib (5mg/kg) (data not shown) and were largely reduced compared to vehicle controls (Fig 4E). In this model, we found that 10 mg/kg was the required dose for effective prophylactic treatment, because 5 mg/kg did not affect the onset or decrease severity of cGVHD compared to the vehicle control group (S4 Fig).

### Ibrutinib improves survival and clinical outcome in the B6→BALB/c model of cGVHD

**Fig 5:**
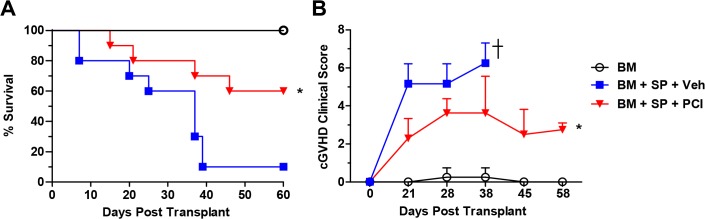
Ibrutinib improves survival and clinical score. Lethally irradiated BALB/c recipients were transplanted with TCD-BM (5 x 10^6^ per mouse) from B6 donors with or without whole splenocytes at a dose of 1–2 x 10^6^ per mouse. Groups either received no treatment (n = 4), daily oral gavage of vehicle (n = 10), or Ibrutinib 10 mg/kg (n = 11) 2–4 hours before BMT and continued for 4 weeks. Survival rate (A) and cGVHD clinical scores (B) were shown from two replicate experiments combined. Asterisk indicates statistical significance between vehicle treatment and Ibrutinib treatment groups: *p<0.05. A one-tailed student *t* test was used in (B).

We then used a recently adapted murine model that recapitulates a transition from aGVHD to a scleroderma-like form of cGVHD with salivary gland involvement and serum antibodies. By transferring a low dose of whole splenocytes from B6 donors into conditioned BALB/c recipients, cGVHD is mainly mediated by *de novo* developed donor-type CD4^+^ T cell and B cells [5]. This model demonstrated the most severe symptomology of cGVHD including severe diarrhea, body weight loss, scleroderma, eye inflammation, and reported defective thymic function [5]. Prophylactic administration of Ibrutinib in this model caused significantly increased survival compared to vehicle controls (Fig 5A). The recipients treated with Ibrutinib also displayed a significantly reduced clinical score compared to those with vehicle controls (Fig 5B).

### Ibrutinib reduces donor T-cell pathogenicity after allo-BMT

**Fig 6:**
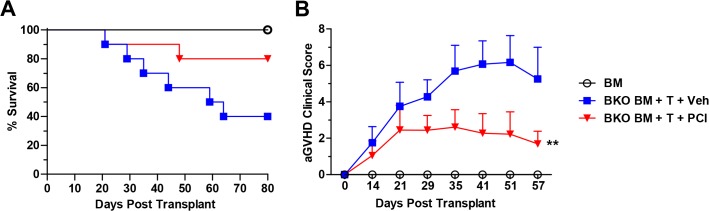
Ibrutinib improves survival and clinical manifestations of cGVHD independent of B cells. Lethally irradiated BALB/c recipients were transplanted with TCD-BM (5 x 10^6^ per mouse) plus purified T cells (0.5 x 10^6^ per mouse) from B6.129S2-*Ighmtm1Cgn*/J (B-cell deficient; BKO) donors, and received daily oral gavage of vehicle (n = 10), or Ibrutinib at 10 mg/kg (n = 10), lasting 4 weeks. Bone marrow alone controls received TCD-BM (5 x 10^6^ per mouse) from WT B6 mice without treatment (n = 4). Animals were monitored for survival (A) and aGVHD clinical score (B) for 80 days. Data are shown from two replicate experiments combined. Asterisk indicates statistical significance between vehicle treatment and Ibrutinib treatment groups: **p<0.01.

Given the inhibitory effect on both BCR and TCR signaling [17], we sought to determine the ability of Ibrutinib to target donor T cells in addition to donor B cells by using a well-established aGVHD model, B6**→**BALB/c, [22, 29, 30], where donor T cells are the culprits of disease. To further exclude the involvement of donor B cells, the recipient mice treated with vehicle or Ibrutinib were transplanted with TCD-BM plus purified T cells from B-cell deficient donors (BKO B6) or from WT B6. The clinical score of the recipients treated with Ibrutinib was significantly decreased throughout the 80-day monitoring period compared to those of vehicle controls (Fig 6B); the same trend was observed 14 days after BMT (D in S5 Fig). Notably, survival was largely improved, although not significantly in Ibrutinib recipients (Fig 6A). In the B6→BALB/c model of aGVHD, we found that 14 days after transplant both CD4^+^ and CD8^+^ T cells expressed significantly higher levels of surface β1 Integrin (CD29) in the recipients treated with Ibrutinib (C in S5 Fig), which was associated with significantly better clinical score (D in S5 Fig), and body weight maintenance (data not shown) when compared to the vehicle group.

## Discussion

It has been shown that cGVHD development is correlated with ITK and BTK expression, as recipients given ITK deficient or X-linked immunodeficiency (XID) splenocytes lacking functional BTK do not develop cGVHD after allo-BMT [31]. However, aside from decreasing B cell and T helper cell activation, various biological mechanisms downstream of ITK and BTK inhibition in the context of aGVHD and cGVHD have not been characterized and are not well understood. Furthermore, other than B and T cells, BTK is also highly expressed on other cells in the hematopoietic lineage such as dendritic cells (DCs), mast cells, and macrophages [32–34]. Genetic BTK deficiency on murine mast cells was shown to hinder production of pro-inflammatory cytokines such as IL-12, TNF-α, and IL-6 [32] suggesting an important link to the involvement of BTK in GVHD.

Here, we show that both BTK and ITK are therapeutic targets, that when inhibited by Ibrutinib, allow for reduced induction or pathogenesis of GVHD in several different mouse models of cGVHD as well as two different models of aGVHD. Prophylactic administration of Ibrutinib can improve survival in two mouse models reflecting cGVHD, including DBA/2 **→** BALB/c (Fig 1A), and B6 **→** BALB/c (Fig 5A), and reduce clinical manifestations in all four models of cGVHD tested (Figs 1B, 2E, 3, 4 and 5B).

Donor B cells can produce pathogenic antibodies within the germinal center of lymphoid organs that are required for tissue inflammation and cGVHD development [6, 7]. Donor B cells can also function as antigen presenting cells (APCs) that facilitate the expansion of pathogenic CD4^+^ T cells [35]. We found that Ibrutinib was able to significantly reduce the amount of proliferated donor B cells, and levels of serum IgG and IgG2a autoantibodies post-BMT (Figs 2 and 3), suggesting that BTK inhibition by Ibrutinib primarily affects B cell function, which could prevent cGVHD or at least delay the disease onset. It has been reported that both CD40 and CD86 costimulatory molecules are necessary for antigen presentation and play critical roles in cGVHD pathogenesis [28, 36], and we observed significant reduction of CD40 and CD86 expression on donor B cells, which may be one of the mechanisms for the effectiveness of Ibrutinib against cGVHD in the models tested (Fig 2C). Notably, when B-cells and T-cells are both in the graft, we did not observe significant change on donor T-cell proliferation upon Ibrutinib treatment in the DBA/2 **→** BALB/c model (S2 Fig). This may indicate a dominant inhibitory effect of Ibrutinib on BTK signaling over ITK signaling, or ITK is not absolutely required for T-cell activation under the development of cGVHD. On the other hand, Ibrutinib was effective in mitigating the clinical manifestations of aGVHD in recipients using two separate models, one of which B cells were essentially absent in the system (Fig 6), indicating the therapeutic effect of ITK inhibition by Ibrutinib that has been suggested by other studies [17]. Additionally, we found that in the B6**→**BALB/c model of aGVHD, Ibrutinib treated recipients showed a significant decrease in the percentage of CD4^+^ T cells in the liver compared to vehicle controls (A, B in S5 Fig). This change in T-cell percentage was accompanied by a significantly elevated population of CD4^+^ T cells in the spleen of the recipients treated with Ibrutinib when compared to those with vehicle controls, suggesting a change in migratory status of these T cells (B in S5 Fig). We also found that both CD4^+^ and CD8^+^ T cells in the recipients treated with Ibrutinib expressed significantly more surface β1 Integrin compared to vehicle controls (C in S5 Fig). It has been demonstrated previously that down-regulation of splenic surface β1 Integrin is correlated with increased splenic α4β7 expression and a resulting increase of CD4^+^ T cell homing to the gut and peyer’s patches [37], and β1 integrin activation, which is contingent on ITK activation, facilitates adhesion of T cells to fibronectin [38]. Thus, our data suggests that Ibrutinib can affect the migratory and or adhesion status of CD4^+^ T cells into GVHD target organs such as the liver at least partially mediated by β1 Integrins [39, 40]. Blockade of ITK via Ibrutinib may also affect adhesion properties of donor T cells as evidenced by increased β1 integrin surface expression upon Ibrutinib treatment (C in S5 Fig) A similar phenomenon has already been shown in the clinic, where several patients with CLL being treated with Ibrutinib showed decreased platelet aggregation due to ineffective platelet adhesion mediated by integrins [41].

Surprisingly, the T cell proliferation and activation profile between Ibrutinib and vehicle treated groups in aGVHD, as measured by CFSE dilution, CD25, and CD62L, was similar (data not shown). However, this result confirms a previous finding that ITK is not necessary for T cell activation or proliferation, and that these traits are not impacted by either genetic deletion or pharmacological blockade of ITK [42]. While not affecting T-cell activation, this group also found that genetic or pharmacological blockade of ITK reduced autoreactive T-cell migration into non-lymphoid organs, possibly as a result of altered integrin expression levels [42]. In cGVHD, we also show that prophylactic administration (10 mg/kg) of Ibrutinib was able to restore the absolute B-cell number (Fig 4H) in B10.D2→BALB/c model, as well as the thymic CD4 and CD8 double-positive T cell number in the DBA/2→BALB/c model (B in S3 Fig) reflecting improved immune reconstitution and decreased severity of GVHD pathogenesis [5]. Additionally, the percentage of Tfh cells and B220^lo^CD138^+^ plasma cells were significantly reduced by Ibrutinib treatment (Fig 4E, 4F and 4G), which suggests Ibrutinib is able to alleviate cGVHD possibly by reducing germinal center B cells as well as pathogenic autoantibody producing plasma cells [43]. Furthermore, we found that mice treated with 10mg/kg Ibrutinib had significantly reduced skin damage caused by cGVHD indicated by H&E pathology scoring (Fig 4B and 4D).

Both complementary and controversial to our study, a recent publication elucidated that Ibrutinib is an effective treatment to reverse established cGVHD when administered 25 days post BMT at the dose of 15 or 25 mg/kg/d in C57BL/6→B10.BR or LP/J→C57BL/6 model, respectively [31]. However, these investigators additionally conducted a prophylactic study of Ibrutinib using the models listed above, which were distinct from models of cGVHD that we tested. Citing their clinical score results for one model (LP/J→C57/BL6), and data not shown for the second model (C57BL/6→B10.BR), they concluded that Ibrutinib was ineffective in preventing cGVHD. Contrary to their results, we elucidated that Ibrutinib, when used as a frequent low dose (<15mg/kg/d) prophylactic, was able to effectively prevent cGVHD characterized by significantly improved survival and reduced clinical manifestations in several distinct mouse models, including a recently adapted model by Zeng’s group, a transition from aGVHD to sclerotic cGVHD accompanied by elevated serum autoantibodies against double-stranded DNA [35, 44]. We pointed out within our own study that the balance of Ibrutinib dosage could be critical in order to preserve the prophylactic effect on GVHD, where 5 mg/kg was ineffective at combating GVHD (S4 Fig) but 10mg/kg was able to prevent or reduce GVHD in several mouse models (Figs 1–6). We used oral gavage of Ibrutinib (10mg/kg/d) instead of administration via drinking water (25mg/kg in LP/J→C57BL/6 model) [31], which may potentially increase the oral bioavailability of Ibrutinib at which a lower dose (10mg/kg) of administration could yield a significantly better outcome on cGVHD prevention. We also conducted experiments to test possible differences between our two groups’ results and found that IP injection of Ibrutinib was less effective at preventing cGVHD in our DBA/2→BALB/c model when compared to oral gavage; suggesting frequent low-dose prophylactic oral gavage is the most effective method to prevent and maintain a durable anti-GVHD response (S3 Fig). As detailed in the pharmacokinetic profile of Ibrutinib provided by the FDA, metabolism of Ibrutinib by the liver into a 15x less potent metabolite following IP injection could present one possibility for these differences [45]. It is also worth noting that these investigators concluded that prolonged Ibrutinib administration was required in order to prevent cGVHD relapse [31], whereas in our studies observing 4 models of cGVHD and 2 models of aGVHD, 3–4 week prophylactic oral gavage treatment was sufficient to prevent GVHD and maintain durable anti-GVHD responses.

Taken together, we demonstrate that the prophylactic blockade of BTK on B cells and ITK on T cells, via Ibrutinib, is an effective treatment strategy for several mouse models of cGVHD and two different models of aGVHD. Thus, our study provides the rationale for using Ibrutinib as a prophylactic treatment to prevent or alleviate cGVHD in clinical trials.

## Supporting Information

S1 Fig Ibrutinib completely prevents ascites but requires at least 3 week administration. Development of ascites in all the recipients at 60 days after BMT was monitored and shown in DBA/2→BALB/c cGVHD model (A). The duration of Ibrutinib administration required to prevent cGVHD development was tested by using only 2 week administration (B). Asterisk indicates statistical significance between vehicle treatment (n = 19) and Ibrutinib treatment (n = 19) groups using a two-tailed Student *t* test: ***p<0.001.
(TIF)

S2 Fig Ibrutinib treatment fails to suppress T-cell proliferation. Representative Day 4 post-BMT flow cytometry panels of T-cell proliferation from the spleens of BALB/c recipients injected with 40 x 10^6^ CFSE labeled CD25^-^ splenocytes from DBA/2 donors.
(TIF)

S3 Fig Gavage not IP injection of Ibrutinib prevents cGVHD and reduces thymic damage. Albumin protein measurements of urine from DBA/2→BALB/c recipients either IP injected or orally gavaged with Ibrutinib (A). Asterisk indicates statistical significance between prophylactic Ibrutinib IP injection (n = 5) and Ibrutinib oral gavage (n = 5) groups using a two-tailed Student *t* test: *p<0.05. In a separate experiment under the same conditions, flow cytometric analysis of thymi from BALB/c recipients treated with vehicle or Ibrutinib 28 days post-BMT showing the CD4^+^CD8^+^ T-cell population (B). Asterisk indicates statistical significance between vehicle treatment (n = 5) and Ibrutinib treatment (n = 5) groups using a two-tailed Student *t* test: *p<0.05.
(TIF)

S4 Fig cGVHD was not prevented by 5mg/kg dose of Ibrutinib Clinical scores (A) and skin histology scores (B) are shown on the recipients treated with vehicle or 5mg/kg Ibrutinib in the B10.D2→BALB/c model. (TIF)

S5 Fig Ibrutinib reduces CD4^+^ T cell homing to liver and increases β1 Integrin expression. Representative Day 14 post-BMT flow cytometry panels from recipient spleens and livers of in the B6→BALB/c model of aGVHD (A). BM source in this experiment was from Ly5.1^+^ donor mice and BM derived CD4^+^ cells were excluded in the analysis by gating on Live/Dead^-^H2k^b+^ population and CD45.1(Ly5.1)^-^CD4^+^ population. Percentage and absolute number of H2k^b+^Ly5.1^-^CD4^+^ cells were shown in the spleen and liver (B). The same gating strategy was used for showing β1 Integrin expression on CD4^+^ and CD8^+^ T-cells (C). Clinical score of the recipients was measured on Day 13 post-BMT (D). Asterisk indicates statistical significance between vehicle treatment and Ibrutinib treatment groups using a two-tailed Student *t* test: ***p<0.001, **p<0.01. In liver section of panel B, Asterisk indicates statistical significance between vehicle treatment (n = 5) and Ibrutinib treatment (n = 4) groups using a one-tailed Student *t* test: *p<0.05.
(TIF)
