# Supplementary figures and images for: Inhibition of BTK and ITK with Ibrutinib Is Effective in the Prevention of Chronic Graft-versus-Host Disease in Mice
Source: PLoS One. 2015 Sep 8;10(9):e0137641. doi: 10.1371/journal.pone.0137641 (PMC4562702; doi:10.1371/journal.pone.0137641)

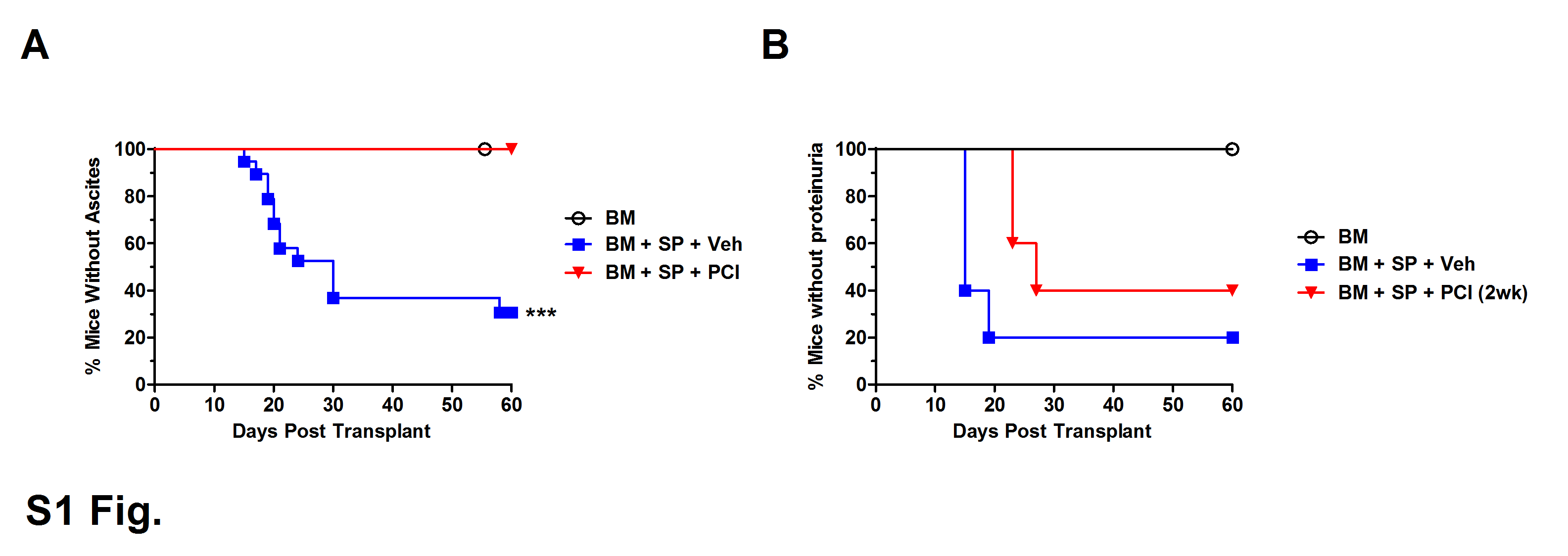

Supplement: S1 Fig — Development of ascites in all the recipients at 60 days after BMT was monitored and shown in DBA/2→BALB/c cGVHD model (A). The duration of Ibrutinib administration required to prevent cGVHD development was tested by using only 2 week administration (B). Asterisk indicates statistical significance between vehicle treatment (n = 19) and Ibrutinib treatment (n = 19) groups using a two-tailed Student t test: ***p<0.001. (TIF) [file pone.0137641.s001.tif]

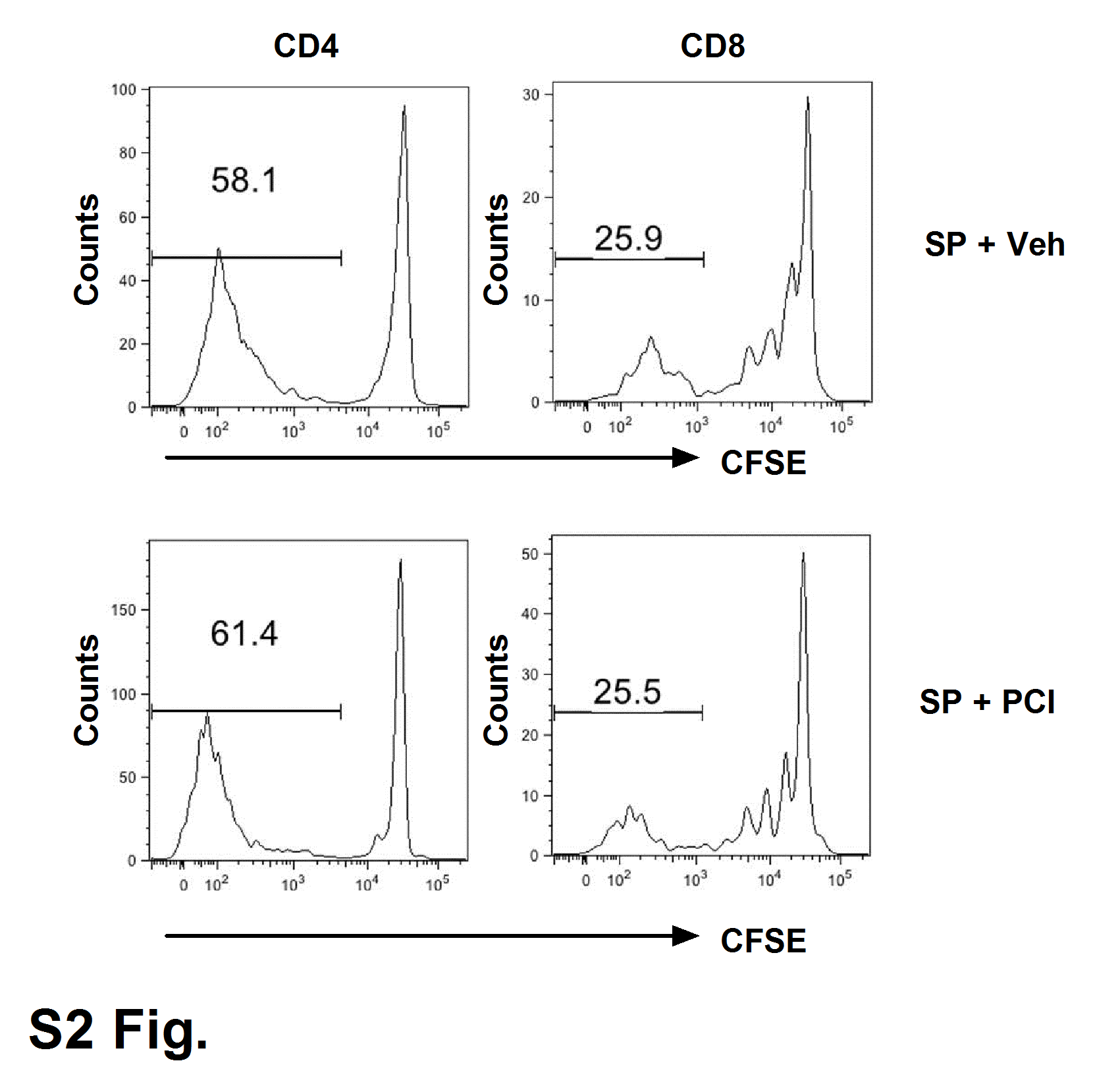

Supplement: S2 Fig — Representative Day 4 post-BMT flow cytometry panels of T-cell proliferation from the spleens of BALB/c recipients injected with 40 x 106 CFSE labeled CD25- splenocytes from DBA/2 donors. (TIF) [file pone.0137641.s002.tif]

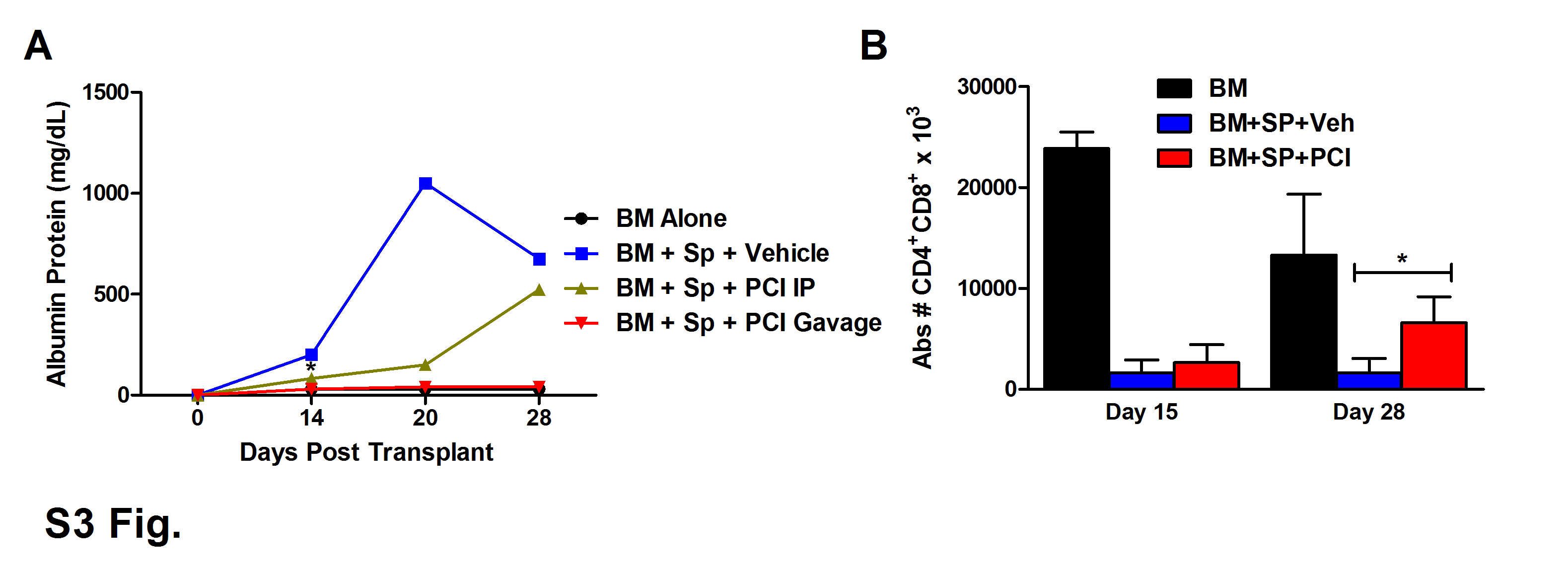

Supplement: S3 Fig — Albumin protein measurements of urine from DBA/2→BALB/c recipients either IP injected or orally gavaged with Ibrutinib (A). Asterisk indicates statistical significance between prophylactic Ibrutinib IP injection (n = 5) and Ibrutinib oral gavage (n = 5) groups using a two-tailed Student t test: *p<0.05. In a separate experiment under the same conditions, flow cytometric analysis of thymi from BALB/c recipients treated with vehicle or Ibrutinib 28 days post-BMT showing the CD4+CD8+ T-cell population (B). Asterisk indicates statistical significance between vehicle treatment (n = 5) and Ibrutinib treatment (n = 5) groups using a two-tailed Student t test: *p<0.05. (TIF) [file pone.0137641.s003.tif]

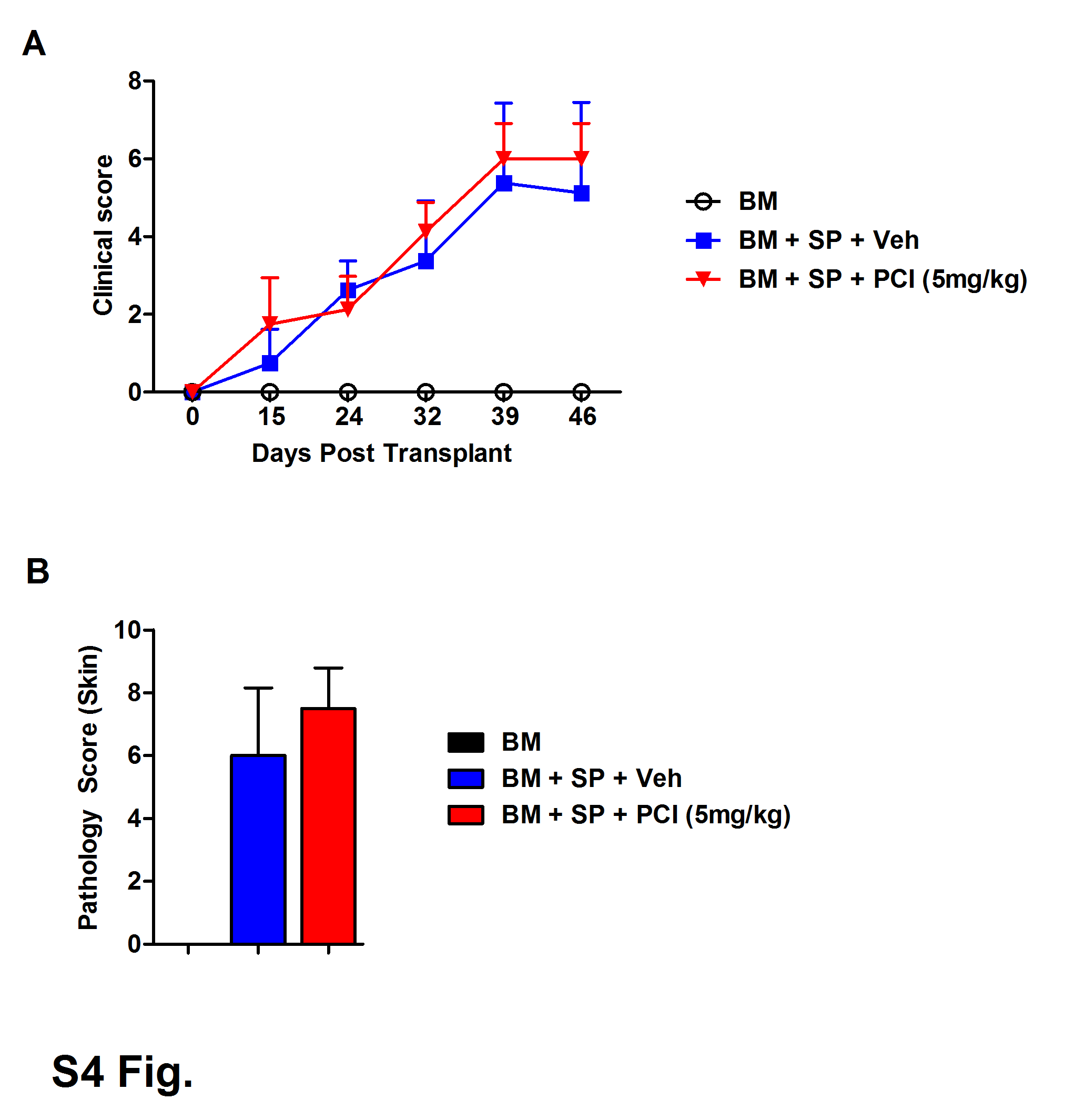

Supplement: S4 Fig — (TIF) [file pone.0137641.s004.tif]

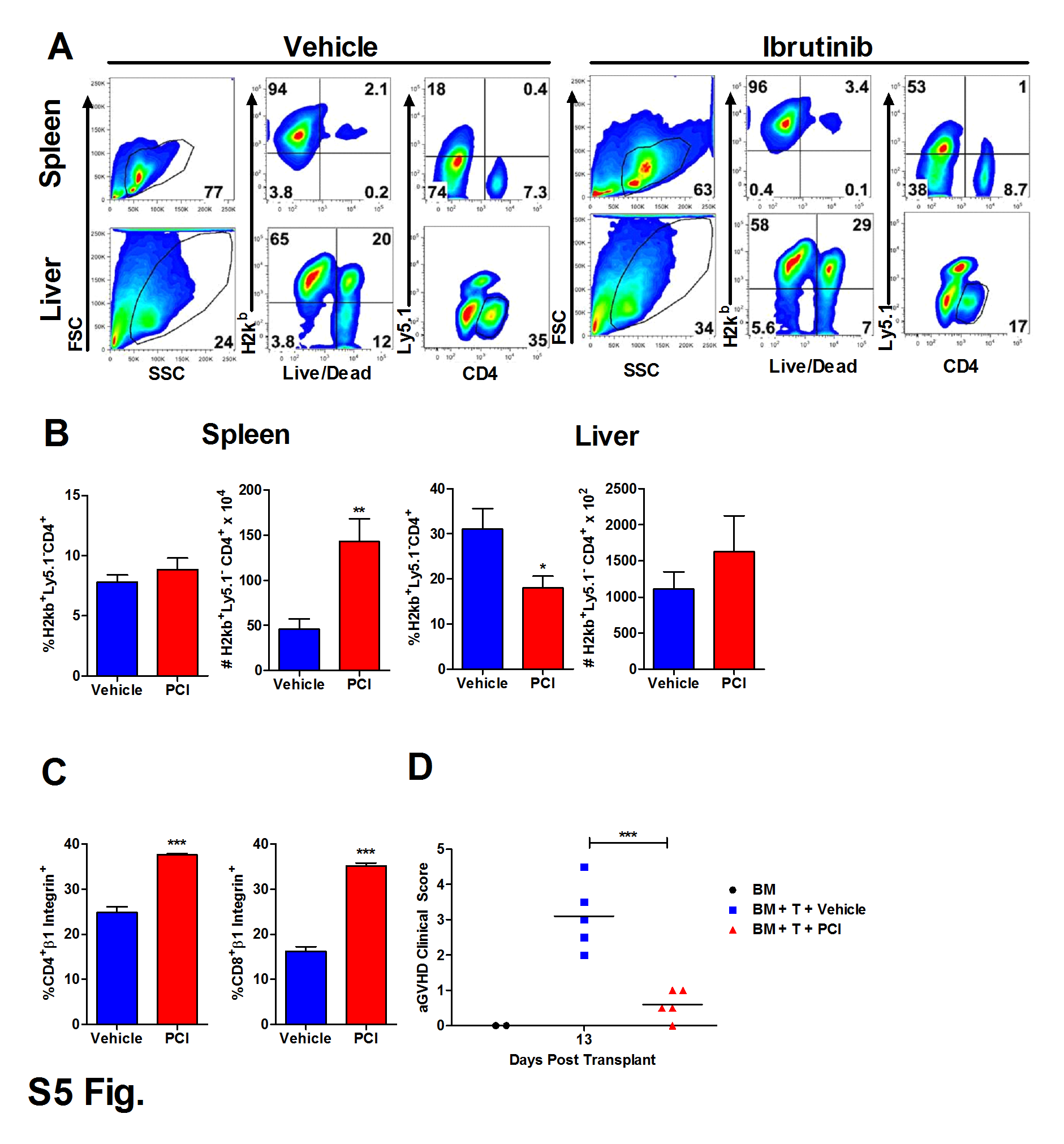

Supplement: S5 Fig — Representative Day 14 post-BMT flow cytometry panels from recipient spleens and livers of in the B6→BALB/c model of aGVHD (A). BM source in this experiment was from Ly5.1+ donor mice and BM derived CD4+ cells were excluded in the analysis by gating on Live/Dead-H2kb+ population and CD45.1(Ly5.1)-CD4+ population. Percentage and absolute number of H2kb+Ly5.1-CD4+ cells were shown in the spleen and liver (B). The same gating strategy was used for showing β1 Integrin expression on CD4+ and CD8+ T-cells (C). Clinical score of the recipients was measured on Day 13 post-BMT (D). Asterisk indicates statistical significance between vehicle treatment and Ibrutinib treatment groups using a two-tailed Student t test: ***p<0.001, **p<0.01. In liver section of panel B, Asterisk indicates statistical significance between vehicle treatment (n = 5) and Ibrutinib treatment (n = 4) groups using a one-tailed Student t test: *p<0.05. (TIF) [file pone.0137641.s005.tif]
